# Rapid Surface Enhanced Raman Scattering (SERS) Detection of Sibutramine Hydrochloride in Pharmaceutical Capsules with a β-Cyclodextrin- Ag/Polyvivnyl Alcohol Hydrogel Substrate

**DOI:** 10.3390/s17071601

**Published:** 2017-07-10

**Authors:** Lei Ouyang, Zuyan Jiang, Nan Wang, Lihua Zhu, Heqing Tang

**Affiliations:** 1 School of Chemistry and Chemical Engineering, Huazhong University of Science and Technology, Wuhan 430074, China; leiouyang@hust.edu.cn (L.Q.); jzy15155029852@163.com (Z.J.); nwang@hust.edu.cn (N.W.); 2 Key Laboratory of Catalysis and Materials Science of the State Ethnic Affairs Commission and Ministry of Education, College of Resources and Environmental Science, South Central University for Nationalities, Wuhan 430074, China

**Keywords:** sibutramine hydrochloride, Surface Enhanced Raman Scattering, β-cyclodextrin, polyvinyl alcohol

## Abstract

Sibutramine hydrochloride (SH) is a banned weight-loss drug, but its illegal addition to health products is still rampant. This suggests a very urgent need for a fast and precise detection method for SH. Surface Enhanced Raman Scattering (SERS) is a promising candidate for this purpose, but the weak affinity between SH and bare metal limits its direct SERS detection. In the present work, β-cyclodextrin was capped in situ onto the surface of Ag nanoparticles to function as a scaffold to capture SH. The obtained Ag nanoparticles were encapsulated into polyvinyl alcohol (PVA) to fabricate a SERS active hydrogel with excellent reproducibility. A facile SERS strategy based on such substrate was proposed for trace SH quantification with a linear range of 7.0–150.0 µg·mL^–1^, and a detection limit low to 3.0 µg·mL^−1^. It was applied to analyze seven types of commercial slimming capsules with satisfactory results, showing good prospect for real applications.

## 1. Introduction

Sibutramine hydrochloride (SH) was a commonly used drug for the treatment of obesity. It could play an significant role in the inhibition of serotonin and norepinephrine reuptake, and makes the body produce heat, hence promoting the consumption of fat tissue [1,2,3,4]. Its good performance in reducing weight made it one of the most adulterated anorexic drugs used in slimming pills [5,6]. However, some serious side effects have been noticed, such as palpitations, chest pains, insomnia [7,8], and even the risk of increasing serious cardiovascular disease [9]. Since October 2010, the suspension of its marketing authorization has been recommended by the U.S. Food and Drug Administration (FDA) (FDA website, http://www.fda.gov/Drugs/DrugSafety/ucm228746.htm). However, its illegal addition to consumer healthcare products is still rampant, thus it is very urgent to develop rapid and precise methods for SH detection.

Several methods have been proposed for the determination of SH and its metabolites. Maluf et al. proposed a quantitative UV-Vis spectrophotometric method for the analysis of SH in aqueous solutions based on its absorption at 223 nm, which was conceptually simple to perform but usually suffers from serious interference from other coexisting components [10]. Phattanawasin et al. developed a thin layer chromatography (TLC)-image method for the quantification of SH adulteration, which was economic but has limited sensitivity [11]. Chorilli et al. described a high performance liquid chromatography (HPLC)-based method for the determination of SH, which provided a linearity range of 4.5–19.5 µg·mL^−1^ [12]. Liquid chromatography-mass spectrometry (LC-MS) and gas chromatography-mass spectrometry (GC-MS) with better sensitivity have also been applied to the quantitative analysis of SH [13,14,15,16]. These methods are highly sensitive and specific, but the required analytical instruments are quite expensive. These methods also have disadvantages of complicated preliminary treatment and time-consuming analysis process, being unfavourable for fast identification. Therefore, it is necessary to develop simple and rapid detection methods for the fast identification and quantification of SH.

As one of most promising methods for fast analysis, surface enhanced Raman scattering (SERS) has diverse applications in the field of food safety and the detection of illegal drug addition [17,18]. A TLC-SERS method was proposed for rapid detection of ephedrine and its analogues which have been used as adulterants in slimming dietary supplements [19]. Zhang et al. used SERS (with nano- silver as substrate) to analyze drugs illegally added into Chinese traditional patent medicines [20]. Trace thiophanate-methyl and the metabolite carbendazim have been analysed in red bell peppers by a SERS imaging technique [21]. The performance of SERS methods is highly dependent on the substrate used [22,23]. Early studies on substrate fabrication mainly involved metal colloids, such as nanospheres [24], nanorods and nanowires [25]. These substrates have good SERS activity, but they also have the disadvantage of easy aggregation which leads to fairly poor reproducibility. Therefore, two dimensional films and gelatins have been used as supports to fix free metal colloids. For example, nanoblocks can be arranged in an orderly way on a filter paper [26], and polyvinyl alcohol (PVA) capsules of uniformly-distributed Ag NPs have been proposed to ensure homogenous SERS responses [27,28]. Such structures can fix NPs and avoid their uncontrollable aggregation, ensuring the reproducibility for SERS detection. The metal gelatin can also harvest plasmonic effects between NP coupling in three dimensional volume, resulting in stronger Raman enhancement [28]. Besides the enhanced ability of the substrate, the interaction between the analyte and the substrate is also very important since only the molecules that come close to the substrate surface could be enhanced. Indeed, our preliminary experiments demonstrated that SH yielded very weak SERS responses, because of the weak interaction between SH and Ag NPs.

In order to promote the SERS performance of analytes (such as SH) without strong substrate interactions, some functional molecules such as graphene oxide may be used as a scaffold to encapsulate such targets [29]. β-Cyclodextrin (β-CD) is one of the functional modifiers used for such applications. β-CD is a cyclic oligosaccharide composed of seven glucopyranose units with a relatively hydrophilic surface and a hydrophobic central cavity [30]. We also noticed that there was host-guest interaction between SH and β-CD, which had been confirmed by many methods such as differential scanning calorimetry (DCS), X-ray powder diffraction (XRD), proton nuclear magnetic resonance spectroscopy (^1^H-NMR) studies and capillary electrophoresis (CE) in earlier studies [31]. Simultaneously, β-CD could reduce metal salts to zero-valence metal through its hydroxyl groups under heat and alkaline conditions [32,33]. The bifunctional role of β-CD might enhance the performance of the affinity of SH to Ag NPs. Here, we propose β-CD modified Ag NPs to detect SH with the aid of a PVA hydrogel. In our previous work, an Ag NP encapsulated PVA substrate was demonstrated to have excellent homogeneity and reproducibility [28]. Its self-standing structure also made it more reliable and versatile in actual analyses. In the present work, by combining a PVA hydrogel and functional β-CD-Ag together, we prepared a β-CD-Ag/PVA hydrogel substrate for fast identification and trace quantification of the SH illegally added to slimming capsules. Both good sensitivity and reproducibility were obtained with the ability to detect SH in the real samples, which showed good prospects for fast and reliable detection of SH in real applications.

## 2. Experimental

### 2.1. Materials

Polyvinyl alcohol (PVA, polymerization degree = 1750 ± 50), silver nitrate (AgNO_3_), nitric acid (HNO_3_), sodium hydroxide (NaOH), and β-CD were purchased from Sinopharm Chemical Reagent Co., Ltd (Shanghai, China). The reagents were of analytical grade. The SH reference standard and the related pharmaceutical capsules were obtained from the National Institute for the Control of Pharmaceutical and Biological Products (Beijing, China). Deionized water was used in all experiments.

### 2.2. Preparation of β-CD-Ag Sol

β-CD (0.01 mol·L^−1^, 3 mL) and NaOH (0.1 mol·L^−1^, 2 mL) were added into distilled water (24.7 mL). The mixed solution was heated to 60 °C with stirring in a water bath, and then AgNO_3_ solution (0.1 mol·L^−1^, 0.3 mL) was quickly added into the mixed solution. After incubating for 1 h, the colour of the solution changed from colourless to yellow, signaling that the Ag ions were reduced to Ag NPs. The obtained NPs were centrifuged at 12,000 rpm for 3 min and washed two times with distilled water. The concentration of obtained the β-CD-Ag NPs was assumed to be 1 mmol·L^−1^ on the assumption that all the initial added Ag^+^ had been reduced to Ag NPs.

### 2.3. Preparation of β-CD-Ag/PVA Gel Substrate

In order to look into the influence of the density of Ag NPs to its SERS performance, the density of encapsulated β-CD-Ag NPs in PVA was adjusted through tuning the dosed NPs concentration. Different concentrations of β-CD-Ag NPs were obtained by centrifuging (12,000 rpm, 3 min) and redispersing. Concentrated Ag NPs from 1 to 9 mmol·L^−1^ were obtained. Then PVA powder (3 g) was dissolved in the concentrated β-CD-Ag sol (17 mL) at 90 °C under stirring to obtain of the β-CD-Ag/PVA mixed sol. The sol was added to a 96-well plate for shaping. The sol was then frozen at −18 °C for 2 h. After being thawed, the obtained hydrogel was ready for use. The Ag/PVA hydrog1el was fabricated as reference, with an in situ reduction procedure based on our earlier work [28]. PVA hydrogel was also synthesized without Ag ion addition.

### 2.4. Characterization

The morphology of the β-CD-Ag NPs was observed with a transmission electron microscope (TEM, FEI Tecnai G20, Hillsboro, OR, USA), scanning electron microscopy (SEM) was performed on a SU8000 FE-SEM instrument (Hitachi, Tokyo, Japan) and the particle size distribution was obtained with a zeta/size analyzer (Nano-ZS90, Malvern, Malvern, United Kingdom). UV-Vis absorption spectra were recorded with an Evolution 201UV-vis spectrometer (Thermo Scientific, Waltham, MA, USA).

### 2.5. Preparation of Standard and Sample Solutions

SH (0.100 g) was dissolved in distilled water (100 mL) to obtain a stock standard solution of 1000 µg·mL^−1^. It was then diluted with water to the different target concentrations. For preparation of real sample solutions, the net contents in the pharmaceutical capsules were taken out and mixed. Part of the mixture was accurately weighed and dissolved in 0.01 mol·L^−1^ HNO_3_ (10 mL) of because of better solubility of SH in acid [34]. Then the mixture was placed in a beaker and sonicated for 30 min at 25 °C. The solution was filtered by a vacuum suction filter device and the supernatant was diluted to 100 mL.

### 2.6. Adsorption Capacity Evaluation

One piece of PVA hydrogel, β-CD-Ag/PVA hydrogel and Ag/PVA hydrogel with the same mass was put into the SH solution with a certain concentration. After being adsorbed for different time intervals, 200 µL of the solution was taken out and filtered for HPLC analysis to determine the concentration of the residue after adsorption. The adsorption capacity was calculated based on the mass of each substrate.

### 2.7. SERS Analysis

A piece of β-CD-Ag/PVA hydrogel was first put into the solution that needs detection (1 mL) for 30 min. Then the substrate was taken out and subsequently subjected to Raman analysis. Raman spectra were measured on a confocal Raman Microscope (DXR, Thermo Fisher Scientific, Waltham, MA, USA). The 532 nm laser was used throughout the experiment with 3.0 mW power. The exposure time was 2 s with 10 scans. For each SERS measurement, we detected ten different positions on one piece of substrate, and their average spectra was used for analysis. We use 900 grooves/mm for detection, and the laser were focused on the surface of the substrate with the confocal Raman instrument.

### 2.8. HPLC Analysis

HPLC analysis of SH was performed on a 1260 LC system (Agilent, Santa Rosa, CA, USA) coupled with an Agilent diode array detector (DAD). A reversed-phase Spherisorb C_18_ column (150 mm × 4.6 mm i.d., 5 μm) was used. The mobile phase was phosphate buffer solution (pH 3) -methanol (40:60, v/v). The flow rate was 1.0 mL min^−1^, the injection volume 20 μL and the column temperature was maintained at 35 °C. The absorbance detector was set at 223 nm for SH detection.

### 2.9. Theoretical Calculations

Raman spectra calculations were performed with the Gaussian 09 software package. The density functional theory (DFT) method with the Becke’s 3 parameters and Lee-Yang-Parr’s nonlocal correlation functional (B3LYP) was used. The basis sets for C, N and H were 3-21G (d) with diffuse function and for Ag, LanL2DZ basis set was applied.

## 3. Result and Discussion

### 3.1. SERS Detection of SH by β-CD-Ag/PVA Substrate

**Figure 1:**
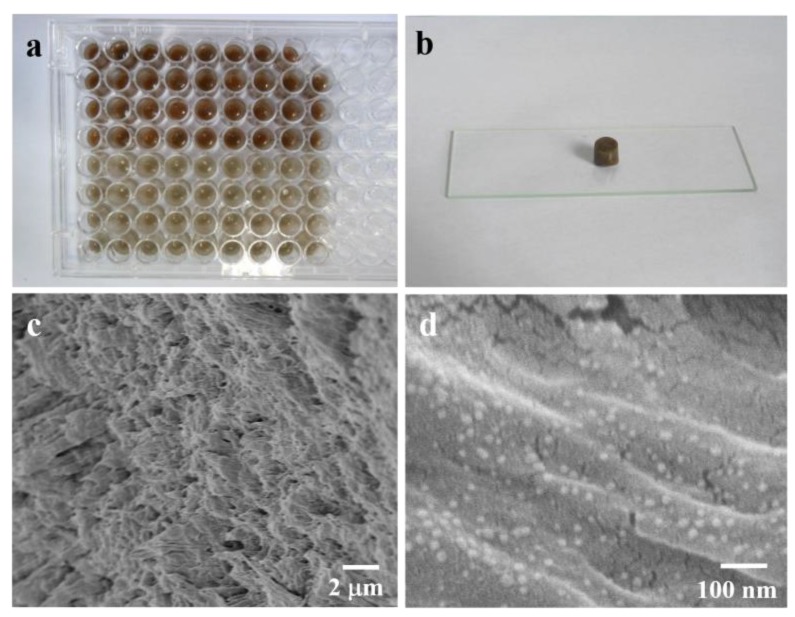
Characterization of the β-CD-Ag/PVA substrate. (**a**) Photograph of the β-CD-Ag/PVA sol before hydrogel formation; (**b**) Photograph of the β-CD-Ag/PVA hydrogel; (**c**,**d**) SEM images of the β-CD-Ag/PVA hydrogel at different magnification.

It is known that β-CD has good recognition ability for SH [30]. It is also reported that the PVA hydrogel structure have the advantage of making use of the three dimensional effective space to enhance SERS sensitivity. Such structures can also inhibit the uncontrollable aggregation of Ag NPs, which would contribute to excellent reproducibility [28]. By combining these properties together, a β-CD-Ag functionalized PVA hydrogel was prepared for the SERS detection of SH. As shown in Figure 1a, the in situ reduced β-CD-Ag NPs were dispersed into the PVA sol at 90 °C under stirring, and then added into a 96-well plate for shaping and further cross-linking into hydrogel after gelling. A photograph of the obtained β-CD-Ag/PVA hydrogel is shown in Figure 1b. The SEM characterization of the hydrogel substrate is shown in Figure 1c,d. The hydrogel was porous and the β-CD-Ag NPs were distributed uniformly in its network.

**Figure 2:**
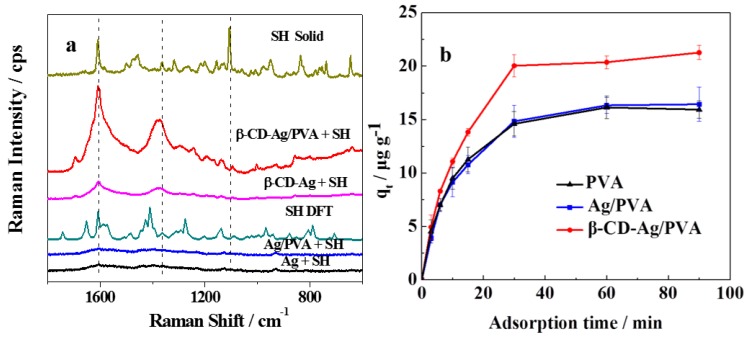
(**a**) Comparison of the SERS performance for SH detection with different substrates; (**b**) Adsorption kinetics of SH on PVA, Ag/PVA and β-CD-Ag/PVA (298 K).

The SERS performance of the obtained substrate for SH detection is shown in Figure 2a. No obvious signal of SH was observed when Ag NPs and Ag/PVA were used as substrates, while several peaks of SH were obtained with the β-CD-Ag NPs and β-CD-Ag/PVA substrate. Characteristic peaks at 1594, 1142, 1098, and 645 cm^−1^ matched well with the Raman spectra of the solid SH powder, and were also consistent to simulation result by DFT calculation. Based on the peak assignment from the calculated data, the peak at 1594 cm^−1^ was attributed to C-C symmetric stretching mode of benzene ring; the peak at 1380 cm^−1^ was signed to skeletal distortion of benzene ring and C-H distortion vibration mode; the peaks at 1142 cm^−1^ and 1098 cm^−1^ belong to C-H distortion vibration mode and 645 cm^−1^ was contributed from C-H distortion vibration mode of benzene ring, respectively. A stronger intensity but similar spectrum was observed after the β-CD-Ag NPs were dispersed into the PVA hydrogel network, which came from the contribution from the three dimensional effective volume as confirmed in our earlier work [28,35]. These results illustrated the good performance of our β-CD-Ag/PVA substrate for the detection of SH.

In order to confirm the promoted SERS performance resulted from the contribution of the modified β-CD, the SH adsorption kinetics of the obtained hydrogels were recorded as shown in Figure 2b. The adsorption capacity of β-CD-Ag/PVA was enhanced by 25% in comparison with Ag/PVA and PVA. Such an obvious enhanced adsorption came from the modification of β-CD which also led to the enhanced SERS performance of our substrate.

### 3.2. Optimization of Substrate Synthesis Conditions

#### 3.2.1. Reaction pH and β-CD Concentration for β-CD-Ag NPs

The SERS performance of NPs is highly dependent on their shape, size and size distribution [36,37]. The size of metal NPs can influence the local electromagnetic mechanism (EM) enhancement, hence influencing their SERS enhancement. In a suitable range, the local EM enhancement increases and more particles scatter through inelastic scattering with increasing particle size [37].

**Figure 3:**
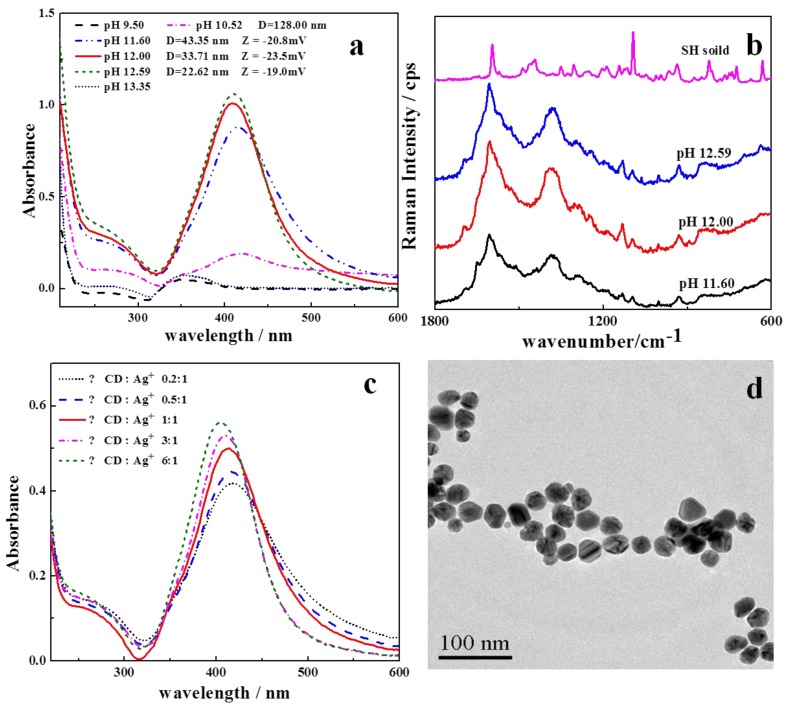
(**a**) UV-Vis spectra of Ag NP suspensions prepared with different synthesis pH values ranging from 9.5 to 13.35; (**b**) SERS spectra of SH on β-CD/Ag NPs synthesized at different pH values; (**c**) UV-Vis spectra of Ag NPs obtained at different ratio of AgNO_3_ and β-CD: 0.2:1, 0.5:1, 1:1, 3:1, 6:1, and 12:1; (**d**) TEM micrograph of β-CD/Ag obtained with AgNO_3_ and β-CD at a ratio of 1:1 ratio.

In the present work, β-CD was used as both a functional scaffold and a reducing agent. β-CD could reduce Ag^+^ to zero-valent Ag through hydroxyl groups under heating and alkaline conditions. The pH may play a key role in the process of the formation and stabilization of Ag nanoparticles [38,39]. Therefore, the influence of reaction pH on the particle size was investigated first. Under the given conditions (reaction volume of 30 mL, 0.01 mol·L^−1^ β-CD 3 mL, 0.1 mol·L^−1^ AgNO_3_ 0.3 mL, reaction temperature 60 °C, reaction time 1 h), the reaction pH was adjusted to 9.5, 10.5, 11, 12.5, and 13 with NaOH, and the obtained NPs were monitored with both UV-Vis spectrometer and laser particle analyzer. As shown in Figure 3a, when the pH was lower than 10.5 or higher than 12.5, no SPR peak of Ag NPs was observed.

Under weak basic conditions (pH < 10.5), the reduction ability of β-CD is too weak to reduce the Ag salt, and in case of a pH higher than 12.5, the strong electrostatic repulsion force between β-CD molecules may prevent the β-CD from densely adsorbing on the surface of Ag NPs, leading to their aggregation. Within the appropriate pH range, namely 10.5–12.5, the SPR peak of Ag NPs could be obtained, which also demonstrated the formation of Ag NPs. Within such a range, the SPR peaks of Ag NPs would blue shift with increasing pH, indicating smaller particle sizes. This tendency was consistent with the results measured by laser particle analyzer as shown in Figure 3a. The SERS performance of the obtained NPs with different particle sizes was shown in Figure 3b. SH could be sensitively detected with the Ag NPs being obtained at pH around 12. According to the result of laser particle analysis, the average particle size of the obtained β-CD-Ag NPs at this pH was 30 nm (Figure 3a).

Similarly, the concentration of β-CD as a reductant also affected the obtained Ag NPs. Here we adjusted the ratio of AgNO_3_ and β-CD to 0.2:1, 0.5:1, 1:1, 3:1, 6:1, and 12:1, and monitored the UV-Vis spectra of the reaction solution. As shown in Figure 3c, as the proportion of β-CD was increased, the intensity of the SPR peaks of Ag NPs increased and the peak position was blue shifted, which indicated a smaller particle size of the Ag NPs. Particles around 30 nm were successfully synthesized with the ratio of 1:1, which were in good agreement with the TEM observation shown in Figure 3d. It was noted that this condition provided the substrate with the best Raman enhancement.

#### 3.2.2. Ag NPs Content in β-CD-Ag/PVA Hydrogel

**Figure 4:**
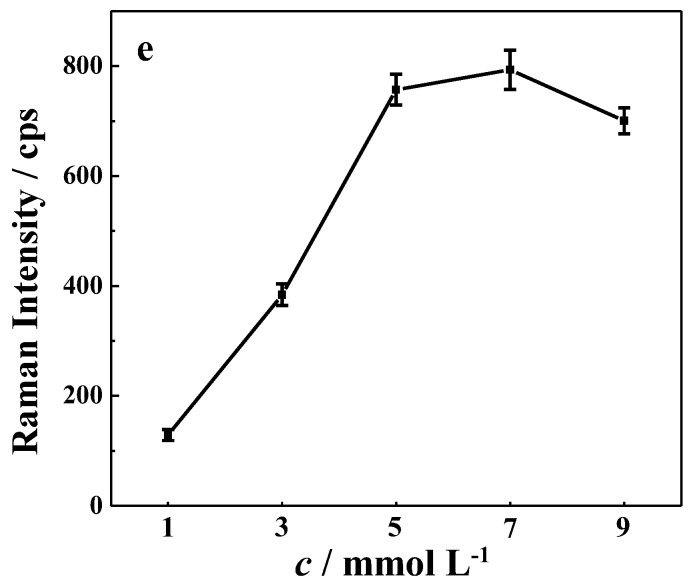
SERS intensity change of SH at 1594 cm^−1^ using β-CD-Ag/PVA with different densities of β-CD-Ag NPs (from 1 to 9 mmol·L^−1^).

PVA has been used as a three dimensional support for β-CD-Ag colloids [35]. The Ag NPs concentration in the PVA gel will affect the distribution of the NPs and hot spots in the hydrogel. Therefore, the Ag NP content in β-CD-Ag/PVA substrates was optimized. By centrifugation, we condensed silver sol and mixed it with PVA to obtain β-CD-Ag/PVA hydrogels with controllable Ag NP content. The content (concentration) of β-CD-Ag NPs was tuned to be 1–9 mmol·L^−1^. As shown in Figure 4, with increasing Ag NPs content in the PVA gel, the SERS activity was first promoted and then remained almost unchanged. Such a phenomenon may result from different numbers of hot spots and effective depth [28]. More hot spots were generated upon increasing the concentration of nanoparticles from 1 to 5 mmol·L^−1^, but when content of NPs was greater than 5 mmol·L^−1^, the light transmittance in the three dimensional β-CD-Ag/PVA hydrogel was inhibited, leading to a decrease in the effective depth and offsetting the influence of nanoparticle concentration increase. The combination of these two factors resulted in the SERS performance being almost the same. Based on the results above, the initial Ag NPs content in β-CD-Ag/PVA was set at 5 mmol·L^−1^.

#### 3.2.3. Optimization of the Detection pH for SH

**Figure 5:**
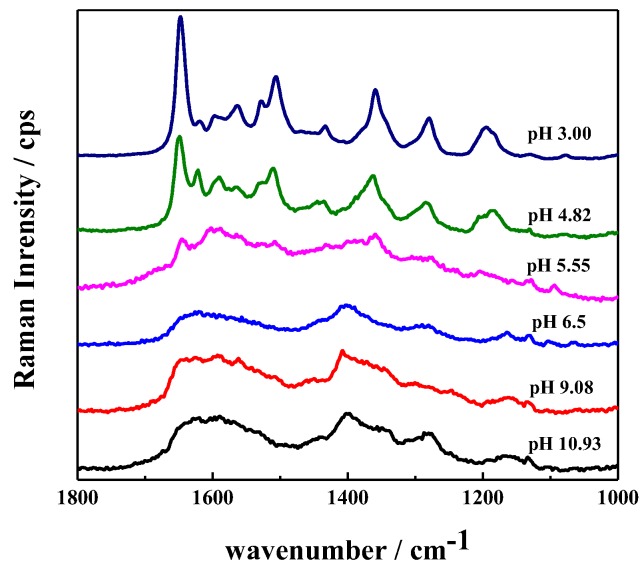
Raman responses of the substrate β-CD-Ag/PVA after being soaked in water at different pH values.

SH is water soluble in acidic solution with a solubility of 2.9 mg mL^−1^ at pH 5.2, but becomes insoluble at pH values higher than 8.5 [12,30]. Therefore, the detection pH should be lower than 8.5. To examine the performance of the obtained hydrogel substrate at different pH values, we soaked the hydrogel in solutions at pH values from 11.0 to 4.8 for 20 min and then recorded their background SERS responses. As shown in Figure 5, at pH 5.55, there were several background interference peaks of the β-CD-Ag/PVA substrate, and their intensity increased with increasing acidity. These background interference peaks might come from the hydrolysis of β-CD under acid conditions [40]. Indeed, the interference peaks of β-CD-Ag/PVA gel at pH 3 were in good agreement with that of β-CD at pH 3 (Figure 5). The β-CD interference peaks disappeared at pH higher than 6.5 due to the inhibition of the hydrolysis. These results illustrated that the hydrogels have good stability in neutral and alkaline conditions. According to the solubility of SH and the stability of β-CD-Ag/PVA gel, we chose pH 6.5 as the detection pH for SH detection.

### 3.3. Analytical Performance for SERS Detection for SH

#### 3.3.1. Linearity

**Figure 6:**
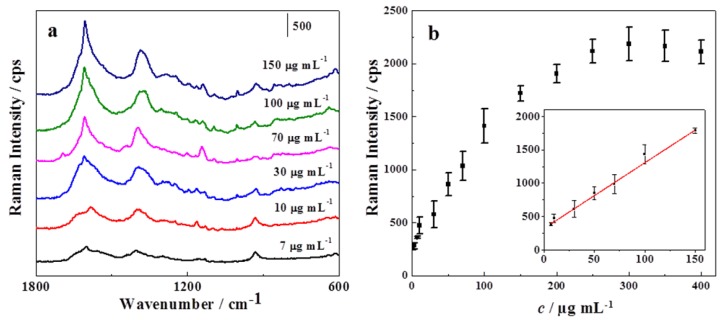
(**a**) SERS spectra of different concentrations of SH; (**b**) A correlation between the Raman intensity at 1594 cm^−1^ and the concentration of SH. The inset in (**b**) showed the linear range within 7–150 µg·mL^−1^ between the intensity at 1594 cm^−1^ and the concentration of SH.

After optimizing the synthesis and detection conditions, the trace detection of SH was carried out on the substrate. Figure 6a shows the SERS spectra of different concentrations of SH.

By choosing the peak intensity at 1594 cm^−1^ as a reference, the correlation between the Raman intensity and SH concentration was obtained as shown in Figure 6b, which had a good linearity over the concentration range of 7–150 µg·mL^−1^ with a regression coefficient R^2^ of 0.986 and a detection limit (LOD) as low as 3 µg·mL^−1^. Such a facile and sensitive method was much better than previously reported work, where the LOD was usually above 20 µg·mL^−1^ [11,13,14].

#### 3.3.2. Selectivity

**Figure 7:**
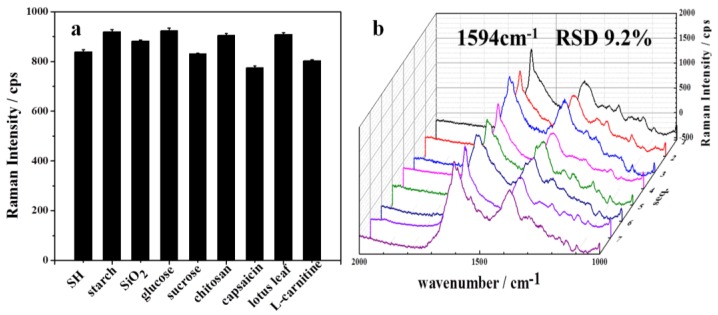
(**a**) Results of confirming the selectivity for SH detection; (**b**) SERS intensity (at the 1594 cm^−1^ peak) of 100 μg mL^−1^ SH on eight batches of β-CD-Ag/PVA substrates, five spots were randomly chosen for each batch of substrate.

The host-guest interaction could ensure the recognition ability of the substrate towards SH. However, we further checked the selectivity of the SERS method on the substrate for real sample detection. The commonly coexisting components in weight loss capsules include excipient materials and effective components. Here we checked the effects of several typical excipient components such as silica powder (SiO_2_), glucose, sucrose, and chitosan, and the effective components such as L-carnitine, capsaicin, and lotus leaf extract. The coexisting components were first mixed with SH, and then the mixtures were subjected to SERS detection. The selectivity of the substrate was evaluated by comparing the results obtained from a 50 µg·mL^−1^ SH standard solution with or without addition of these coexisting components (added concentrations were 50 times their common contents in pharmaceutical capsules). As shown in Figure 7a, little difference was observed after the coexisting components were added (the data was tested by *t*-test), which means that our method has good selectivity for SH.

#### 3.3.3. Reproducibility between Different Batches

In order to prove the reproducibility of our proposed substrate, the SERS response of SH (100 µg·mL^−1^) were obtained for eight different batches of β-CD-Ag/PVA gel. As shown in Figure 7b, the SH Raman spectra from eight batches substrates were similar to each other. The obtained relative standard deviation (RSD) of the intensity at 1594 cm^−1^ was less than 9.2%, indicating good reproducibility of the proposed β-CD-Ag/PVA substrate.

#### 3.3.4. Precision

The precision of our analysis strategy was also evaluated by monitoring its systematic precision, and intermediate precision (both intra-day and inter-day precision). The obtained results were evaluated by the RSD of the peak strength at 1594 cm^−1^. The system precision was analyzed by detecting the same sample for six times. The intra-day intermediate precision was demonstrated by detecting the same sample six times during the same day, while the inter-day intermediate precision was demonstrated by detecting one sample for six times in different days. The SH concentration was controlled at 50 µg·mL^−1^. By recording the peak intensity at 1594 cm^−1^, it was found that the RSD values of the system precision, intra-day precision and inter-day precision experiments were 7.5%, 6.8% and 9.5%, respectively. These results confirmed that the method has a good precision.

#### 3.3.5. Accuracy

The accuracy of the observed results was evaluated by monitoring the recovery (here we defined the recovery to the percentage of the detected mass (or concentration) to its real mass (or concentration) added into the samples), SH standard solution was added into the samples and their SERS response were obtained. Each sample was analysed twice. The results are summarized in Table 1. The observed mean recovery for the compound was 96.7–104.2%. We also conducted a most commonly used HPLC method as contrast. As shown in Table 1, the test results of two methods were consistent with each other, indicating that the accuracy of the developed method is good in quantifying the concentration of SH.

#### 3.3.6. Real Samples Detection

After all the analytical performances were confirmed, we used the established method to analyse practical samples. Several widely used pharmaceutical capsules were obtained from a local pharmacy. Among seven kinds of diet pills, one was detected to contain SH, with a concentration of 270 µg·g^−1^, and the other six kinds of drug showed negative results.

We also added different concentrations SH standard into the negative samples and calculated the recovery. The results are shown in Table 2. These results showed that our method can be used in the fast quantitative determination of practical samples.

## 4. Conclusions

A β-CD-Ag/PVA SERS substrate was successfully synthesized and applied to quantify trace concentrations of SH. The host-guest interaction between β-CD and SH enhanced the detection activity of the substrate for SH. The PVA encapsulating β-CD-Ag NPs substrate offered a good free-standing structure for functional Ag NPs, which can provide a three dimensional substrate with excellent homogeneity and reproducibility. After optimizing the experimental conditions, the proposed method showed good performance for SH detection in the concentration range from 7 µg·mL^−1^ to 150 µg·mL^−1^, the obtained detection limit was low to 3 µg·mL^−1^. The validated method exhibited excellent selectivity, accuracy, and precision for the SH detection. The presented method was also successfully applied into the quantification of SH in real pharmaceutical capsules. Such a rapid and economical method shows good prospects for the fast detection of SH in real samples.

## Figures and Tables

**Table 1:** Detection of positive samples with both SERS and HPLC method. m_s_: the detected mass of SH in capsules sample, m_a_: mass of SH added to capsules, m_d_: the detected mass of SH. ω% represents the recovery of detected SH compared to its real concentration. ϖ% is the mean value of ω% for three times of detection.

m_s_/m_g_	m_a_/m_g_	m_d_/m_g_	ω%/SERS	ϖ%/SERS	RSD%	ϖ%/HPLC
0.504	0.400	0.885	97.9	96.7	4.1	99.3
0.502	0.400	0.860	95.4			
0.523	0.500	1.119	109.4	103.7		98.9
0.582	0.500	1.060	98.0			
0.528	0.600	1.089	96.6	104.2		100.2
0.471	0.600	1.196	111.7			

**Table 2:** Detection results of six negative real samples with SH addition.

Samples	Theoretical/mg	Detection/mg	ω%/SERS	ϖ%/SERS
1	0.500	0.473	94.9	99.5
	0.500	0.548	109.6	
	0.500	0.470	94.1	
2	0.500	0.467	93.3	93.8
	0.500	0.489	97.9	
	0.500	0.452	90.3	
3	0.500	0.471	94.1	96.3
	0.500	0.468	93.5	
	0.500	0.507	101.3	
4	0.500	0.483	96.6	101.1
	0.500	0.483	96.7	
	0.500	0.548	109.5	
5	0.500	0.549	109.8	104.8
	0.500	0.507	101.3	
	0.500	0.516	103.1	
6	0.500	0.484	96.8	95.6
	0.500	0.486	97.1	
	0.500	0.466	93.1	
